# Education Research: A Mixed-Methods Analysis of Journal Club Formats to Enhance Evidence-Based Practice Through Social Cognitive Learning Theory

**DOI:** 10.1212/NE9.0000000000200195

**Published:** 2025-02-11

**Authors:** Katherine A. Fu, Joy M. Chan, Katelyn Stepanyan, Sally Elting, Ashley Manchanda, Alonso Gonzalo Zea Vera, Michelle Vermillion, Holly Wilhalme, Adrienne M. Keener, Roy E. Strowd

**Affiliations:** 1 Department of Neurology, University of California, Los Angeles;; 2 Department of Medicine, University of California, Los Angeles;; 3 Department of Medicine, University of California, San Francisco;; 4 Department of Neurology, Children's National Hospital, Washington, DC;; 5 David Geffen School of Medicine, University of California, Los Angeles;; 6 Department of Medicine Statistics Core, University of California, Los Angeles; and; 7 Department of Neurology, Wake Forest University, Winston-Salem, NC.

## Abstract

**Background and Objectives:**

In an era of rapid advances in research, it is imperative for residents to develop the skills for evidence-based practice. Alternative journal club formats grounded in active learning strategies may be better suited to address this need, although evidence is lacking. A theoretical framework of social cognitive learning theory (SCLT) may provide insight into how journal clubs can be better designed to teach residents how to evaluate research. Using a sequential, explanatory mixed-methods design and SCLT framework, we compared 2 different journal club formats for teaching methodology and clinical application. We also explored neurology resident experiences with both formats using a qualitative approach.

**Methods:**

We conducted 4 alternating journal clubs: 2 active learning and 2 traditional. In the quantitative phase, we compared resident performance on presession and postsession assessments for the 2 different journal club formats. We designed parallel pretest and posttest forms and analyzed the change in scores using a linear mixed-effects model with fixed effects for the test type and pretest score. In the qualitative phase, we explored neurology resident experiences with both formats through the lens of SCLT. We observed the journal club sessions through an ethnographic lens and conducted semistructured one-on-one interviews with residents. The data were mixed during analysis of interview data.

**Results:**

Resident participants (n = 18 active learning, 18 traditional) of each journal club completed pretests (n = 16 active learning, 11 traditional) and posttests (n = 8 active learning, 5 traditional). There were statistically significant increases in total (estimate = 18.03%, SD = 6.9, *p* = 0.028) and clinical application (estimate = 48.40%, SD = 6.6, *p* < 0.0001) pretest and posttest scores with the active learning format. There was no difference in methodology subscores (estimate = 5.84%, SD = 11.8, *p* = 0.63). Regarding the active learning format, observations noted more demonstrations of retention, reproduction, and motivation during the sessions than a traditional format. Interviews highlighted 4 main themes: the burden of preparation, the importance of clinical relevance, preference for organic discussion, and the value of faculty expertise.

**Discussion:**

These results highlight the strengths and disadvantages of a more discussion-based journal club format. Further altering the design to a “no-prep” approach and emphasizing the faculty facilitator's role may further optimize teaching of evidence-based practice.

## Introduction

The rate of development of novel therapeutics in neurology has grown in recent years with a high number of accompanying research studies.^1,2^ As a result, the amount of information that neurology residents are expected to synthesize and integrate into clinical care has grown significantly. The COVID-19 pandemic also promoted a surge in published research of disparate quality, including expedited peer-reviewed and preprint publications.^2,3^ Some of these articles contained flaws,^2^ leading to article withdrawal^4^ and potential dissemination of misinformation through social media.^5^ Thus, the ability to efficiently critically analyze research and apply these findings to clinical care is paramount.

The Accreditation Council for Graduate Medical Education and American Academy of Neurology have recognized the importance of this skill in the Neurology Milestones 2.0, the “Evidence-Based and Informed Practice” subcompetency.^5,6^ While journal clubs have historically been used to teach evidence-based practice, traditional journal club formats have disadvantages such as variable audience engagement and inconsistent preparation.^6^ To address these disadvantages, training programs have studied the possibility of transitioning to an active learning journal club model, in which participants are divided into small groups to lead the discussion of a particular component of the journal article.^6^ This approach integrates the principles of social cognitive learning theory (SCLT), emphasizing Bandura's 4 components of observational learning: attention, retention, reproduction, and motivation.^7,8^ Small-group discussions enhance attention to peers' contributions, encourage retention of material and reproduction of critical analysis skills, and aim to improve motivation for learning through active participation.

Previous work has demonstrated that residents and faculty prefer the active learning model over the traditional model in achieving journal club objectives, understanding content, and feeling comfortable during the discussion,^6,9^ but data beyond satisfaction-level outcomes are lacking. The quantitative phase of this study aims to compare learning outcomes related to principles of research methodology and clinical application of research across traditional and active learning journal club formats. The qualitative phase further explores these findings by examining resident experiences with both formats.

## Methods

We conducted a quasiexperimental, sequential, explanatory mixed-methods study comparing the traditional format with an active learning format. Quantitative data collection involved analysis and comparison of pretests, posttests, and delayed posttests for each journal club session across the 2 formats. There were 2 qualitative components consisting of observations of the journal club sessions and one-on-one semistructured interviews with resident participants.

### Study Setting and Participants

This study was conducted at the University of California, Los Angeles (UCLA) Neurology residency program. Of the 29 neurology residents who were eligible, an average of 18 participated in both active learning and traditional journal clubs (Table 1). The number of resident attendees by training level is presented in Table 1.

**Table 1:** Journal Club Attendance Demographics

Journal club session	PGY-2	PGY-3	PGY-4	Total residents in attendance
#1 (Active learning)	9	6	3	18
#2 (Traditional)	10	5	6	21
#3 (Active learning)	9	4	5	18
#4 (Traditional)	7	4	4	15

### Journal Club Description

Resident facilitators were selected based on their participation in the research special interest track of the residency program. Faculty facilitators were chosen as content experts that aligned with the journal article. Examples of facilitator instructions can be found in Supplemental file 1 for both traditional (eAppendix 1) and active (eAppendix 2) learning.

In the traditional journal club format, a resident facilitator was assigned to present a preselected journal club article. Article preselection enabled the creation of tailored pretest, posttest, and delayed posttest questions. A faculty facilitator was also invited to offer additional comments and expertise. Facilitators were instructed to welcome comments or questions during the journal club itself, while also dedicating at least 10 minutes at the end for questions and discussion.

In the active learning journal club format, the resident facilitator created a discussion guide based on the “active learning” format described in an emergency medicine residency program.^6^ The discussion guide consisted of an article summary and 1–3 discussion questions for each section of the research article (Introduction, Methods, Results, Discussion). To minimize preparation burden, we implemented a jigsaw approach, assigning each postgraduate year class to prepare a specific section of discussion questions. Discussion items were assigned by email to participating residents 1 week before the journal club session in addition to the journal article. During the session, the resident and faculty facilitator guided the discussion as one large group (in contrast to the original format of the original article). Table 2 lists additional similarities and differences between the 2 formats.

**Table 2:** Similarities and Differences Between Traditional and Active Learning Journal Club Formats

Traditional format	Active learning format
Similarities	
Preselected article	
A single resident facilitator and faculty facilitator were identified to lead each session	
Both facilitators provided instructions 1 mo in advance (Supplement 1)	
Hybrid sessions with both in-person and virtual attendance options	
Journal club article was distributed to all residents 1 wk in advance of the session	
Differences	
Resident facilitator created a presentation highlighting relevant aspects of the article	Resident facilitator created a discussion guide, which included a summary of the article and discussion questions
Residents read article as preparation (up to 1 wk in advance)	Residents read article and answered their assigned section questions as preparation (jigsaw approach) (up to 1 wk in advance)
Residents had the opportunity to ask questions both during and at the end of the journal club	Residents provided answers to their respective discussion questions (jigsaw approach)
Faculty facilitator offers expertise and answers questions	Faculty facilitator guides discussion of answers to questions

Four journal club sessions were conducted during the 2022–2023 academic year monthly from January through April of 2023, alternating between active learning and traditional formats.

### Quantitative Methods

In the quantitative phase, our primary objectives were to evaluate whether the traditional or active learning journal club format was more efficient at teaching neurology residents evidence-based practice, defined as principles of research methodology and clinical application of research.

#### Creation of Multiple-Choice Tests

We created multiple sets of article-specific 10-question multiple-choice quizzes to serve as pretest, posttest, and delayed posttest assessments. KF created the initial set of 40 questions, 10 questions for each of 4 journal club articles. To enhance their validity, these were reviewed by a Senior Educational Analyst of the Educational Measurement Unit of the David Geffen School of Medicine (DGSOM) at UCLA (M.V.). Multiple rounds of revisions were applied to this initial set to improve clarity and to ensure demonstrations of knowledge and knowledge application. The questions were piloted with trainees and faculty (J.M.C., A.Z.V., R.S.) for content accuracy and difficulty level to mitigate ceiling effects in the analyses. After revising the questions based on the pilot, the questions were again reviewed before finalization (M.V.). Once this initial set was finalized, we created sister sets of questions to avoid reuse of the same question sets for the pretests and posttests. Therefore, 2 additional sets of 40 questions (80 in total) were created (K.A.F., J.M.C.) and again reviewed by M.V. for accuracy and to ensure that the sister questions were sufficiently similar to the original set, yet different enough to minimize direct recall of previous questions.

#### Administration of Multiple-Choice Tests

The journal club pretests were administered 1–2 days before the journal club, and facilitators were also instructed to allot the first 10 minutes of the journal club session for pretest completion. They were also instructed to allot the final 10 minutes of the journal club for posttest completion. Post-tests were made available for completion up to 2 weeks after the journal club session. Delayed posttests were administered approximately 1 month after the journal club. Each test had 10 questions, and the outcomes were calculated as the percentage correct out of 100%. In addition, each test had 2 types of questions included: (1) research methodology and (2) clinical application. The number of research methodology and clinical application questions alternated between 7 and 3, respectively, and 5 and 5, respectively, with each journal club format (active learning and traditional) having one test of each type. The learning objectives assessed by the multiple-choice tests are listed in Table 3.

**Table 3:** Learning Objectives for Journal Club Curriculum

Research methodology	Clinical application
Recognize the key inclusion and exclusion criteria of participants enrolled in the discussed research study	Demonstrate proficiency in applying the findings of the discussed research article to patient care by successful answering of case-based questions on the postsession test
Discuss the strengths and weaknesses of the study design of the discussed journal club article and evaluate its appropriateness for the research question	
Critique the adequacy of the sample size and the ability of the sampling process used to select a representative sample in the discussed article	
Determine the appropriateness of the statistical analysis methods to the research question and type of variables studied	
Discuss the possible source of bias and limitations of the study	
Determine the external validity (generalizability) of the results of the discussed clinical research article	

### Qualitative Methods

#### Qualitative Observations

We conducted observations of journal club conversations through a focused ethnographic lens to facilitate understanding of social dynamics during journal club sessions and capture elements of SCLT (attention, retention, reproduction, motivation) that were or were not demonstrated during each session (Table 4).^10^ The observer (J.M.C.) then completed the field guide during each journal club. Observations occurred during each of the 40–50-minute-long journal club sessions, documenting resident interactions with each other and the facilitators. Debriefing between the observer and the principal investigator occurred within 24 hours of the journal club session, addending the field guide with additional notes. Field notes were stored in a secure online location.

**Table 4:** Four Components of SCLT and Corresponding Behaviors

Component of SCLT	Examples of behaviors
Attention	- Eye contact with other participants or facilitators - Attentiveness to the presentation - Respectful discussion - Multiple speakers speaking at the same time
Retention	- Participant makes a comment demonstrating a fact read from the article, learned from the presentation, or learned from an earlier point of the discussion - Participant answers one of the discussion questions based on knowledge of the article or presentation
Reproduction	- Participant makes a comment demonstrating critical analysis of the article based on the presentation or the discussion - Participant answers one of the discussion questions by expanding or building on a comment made by another participant - Participant applies what was learned earlier in a journal club session to make a substantial comment
Motivation	- Faculty or resident facilitator praising a substantial comment - Participant praising a previous comment by another participant - Faculty or resident facilitator critiquing a comment from a participant - Participant critiquing a previous comment by another participant

#### Semistructured Interviews

Eligible neurology residents from all stages of training were invited to participate in 30-minute semistructured individual interviews using a purposive sampling approach based on participation. The initial interview guide was developed to pose questions aligned with the elements of observational learning in SCLT. All interviews were conducted over Zoom, recorded, transcribed, and deidentified before analysis. To ensure trustworthiness of data, we implemented member checking, and transcripts were sent to participants for review of accuracy and additional comments.

### Data Analysis

During quantitative data analysis, 3 outcomes were analyzed: (1) the change in the overall score from pretest to posttest, (2) the change in the research methodology subscore from pretest to posttest, and (3) the change in the clinical application subscore from pretest to posttest. The data were analyzed using a linear mixed-effects model of the change in the score from pretest to posttest with fixed effects for journal club format, with the pretest score and test type as covariates. Individual scores for pretests and posttests were linked by unique identifiers, and only scores for which there was a pretest and posttest available were included. A random intercept for subject was used to account for repeated measures. The Satterthwaite approximation was used to estimate the denominator degrees of freedom. All statistical analyses were conducted in SAS version 9.4 (SAS Institute, Cary, NC). *p* Values <0.05 were considered statistically significant.

Qualitative analysis was completed by 2 study investigators (K.S. and K.A.F.) using coding software Dedoose (version 9.0.107; SocioCultural Research Consultants, LLC, Los Angeles, CA). Triangulation of data across observations and semistructured interviews to support credibility and the use of multiple coders to support dependability of data were also performed.^11^ Using a hybrid deductive and inductive approach, initial codes were independently generated from 2 interview transcripts. After reaching consensus over several meetings with both investigators, the initial codebook was generated and applied to additional transcripts with a Cohen kappa coefficient of 0.85. This codebook was then applied to the remaining transcripts, and initial themes were generated using thematic analysis. Themes were iteratively revised until consensus was reached. Interviews were conducted until thematic saturation was achieved.

### Ethical Approval

The institutional review board reviewed this submission, which was deemed certified exempt (UCLA IRB # IRB#22-001247).

### Data Availability

The data that support the findings of this study are available on request from the corresponding author. The data are not publicly available because of privacy and ethical restrictions.

## Results

### Quantitative Analysis

Table 1 presents the number of participants who attended each journal club session, and Table 5 presents the number who completed each pretest and posttest. Completion rates decreased from pretest to posttest for all 4 journal club sessions. Delayed posttests were not included in the analysis because of low-response rates (n ≤ 3).

**Table 5:** Number of Tests Completed

Journal club session	Pretest	Posttest
#1 (Active learning)	16	8
#2 (Traditional)	11	5
#3 (Active learning)	5	4
#4 (Traditional)	6	2

There were statistically significant increases in total score difference (estimate (SE) = 18.03 (6.9); *p* = 0.0278) across pretests and posttests with the active learning format when compared with the traditional format. A subanalysis compared the 2 types of questions included in the tests: (1) research methodology questions and (2) clinical application questions. This subanalysis demonstrated that the significant improvement seen in the active learning format was largely driven by the clinical application questions (estimate (SE) = 48.40 (6.6); *p* < 0.0001). There was no significant difference in the methodology question pretest and posttest scores (estimate (SE) = 5.84 (11.8); *p* = 0.63). Note that estimates are percentages. Table 6 provides the estimated change from pretest to posttest by journal club format using the mean pretest score and test type to calculate the estimates.

**Table 6:** Modeled Estimates of the % Change From Pretest to Posttest by Journal Club Format

Outcome	Active learning Mean (SD)	Traditional Mean (SD)	*p* Value
Total score difference	22.753 (5.613)^a^	4.725 (6.744)	0.0278
Methodology subscore difference	7.052 (8.202)	1.214 (10.061)	0.6296
Clinical application subscore difference	49.719 (5.288)^a^	1.322 (6.374)	<0.0001

a Significant change from pretest to posttest.

### Qualitative Analysis

#### Demonstrations of SCLT During the Journal Club

Observations noted similar demonstrations of attention across both formats, but more consistent demonstrations of retention, reproduction, and motivation in the active learning format. Positive motivating comments were predominant across both formats, but more motivating comments were observed in the active learning than in the traditional format. A greater number of participants engaged in active discussion in the active learning format compared with the traditional format. Around half of all the participants present engaged in discussion during the active learning format while less than a third of participants engaged in discussion during the traditional format.

#### Thematic Analysis

Eight neurology residents completed interviews. Thematic analysis revealed 4 main themes related to the experience of the traditional and active learning journal club formats.

##### Theme 1: The Paradox of Preparation

When asked about challenges of the journal club that impeded learning, residents reported a lack of time to adequately prepare for the session. This lack of preparation time affected their perceived ability to engage and learn in both the active learning and traditional formats.

I think the main challenge just throughout residency in general is finding the time to actually read the papers. I know that there were times where I couldn't read it ahead of time or some of my colleagues couldn't read it ahead of time, and so during the journal club or right before it we were taking couple minutes to try and read the pertinent sections. I think that I'm not sure how we would address that really because I do feel like you really need to read the paper to discuss it. —R5

I just know that if you read the article ahead of time you gain more. I think that is true for sure…when are we gonna give the actual time? We're all working. Are you gonna give 30 minutes before the journal club? Is 30 minutes even enough for some people? Maybe. Or maybe you'll just skim through the abstract and that's good enough…. Giving them the protected time may be helpful. Is that really feasible? I'm not exactly sure. —R8

The excerpts given above demonstrate the perceived importance of pre-reading the article to meaningfully participate. Residents acknowledged that adequate preparation was important, whether it took the form of reading the article beforehand (traditional) or preparing for the discussion questions (active learning). However, given multiple competing professional and clinical commitments of residency training, finding time for preparation was challenging. Although some residents suggested the idea of adding protected time for preparation, they questioned the feasibility of such a strategy.

##### Theme 2: Clinical Relevance Is a Strong Internal Motivator

When discussing residents' motivation to prepare or participate in the journal club, the clinical relevance and topic of the articles emerged as key drivers in motivating residents to engage with research articles.

I think more of finding articles that are really, again, that are really either of hot, current topics, current hot topics in the neurology. That would probably have enhanced my engagement. —R6

The topics were not necessarily ones that I was the most interested in or would pertain to my future practice. —R1

Most residents reported more interest in the clinical implications of research rather than the nuances of research methodology. They also expressed a consistent desire to have more input in selecting a topic that is most relevant to their future subspecialty or area of interest.

##### Theme 3: Passive Listening Predominates in Both Formats, With Inorganic Dialogue Characteristic of the Active Learning Format

When asked to describe their overall participation in the active learning and traditional journal club formats as either more active or more passive, residents acknowledged that they participated less in the traditional journal club model and were mostly engaged in passive listening. However, despite the increase in participant speaking in the active learning model, passive listening remained the predominantly reported experience as well.

(Active learning journal club) “Like I said, we just were going around and then reading the important parts and prompt. Other than that, I guess—my interaction specifically wasn't there too much because, like I said, I wasn't participating—I was mostly listening to the problems. Then when the other residents would say something, again, the leading—the peer leader would chime in.” —R5

Based on the content of the interviews, passive listening seemed to predominate because of the inorganic nature of discussion in the active learning format. Although more individuals were expected to speak during their assigned section, this occurred in a heavily scripted manner and did not necessarily lead to active participation in the rest of the discussion.

##### Theme 4: Faculty Modeling and Development Are Highly Valuable in the Learning of Evidence-Based Practice

When asked to reflect on aspects of journal club that enhanced their learning or affected how they understand research methodology, faculty participation stood out as a recurrent source of highly valued instruction during journal club sessions.

The faculty member had some educational points on study design, which I wasn’t aware of before, in terms of how to just put together the methods and decide on the methods. —R1

Learning arises from faculty modeling of evidence-based practice, specifically how they interpret research data and apply these data to clinical practice. The content of faculty expertise included offering commentary on study methodology, providing helpful summaries, and making connections to clinical practice.

#### Integration

On mixing the quantitative and qualitative results, we found that there was confirmation of both data sets. There were no differences in methodology subscores across the 2 different formats and few demonstrations of changes in understanding of research methodology mentioned during interviews with little motivation to learn about this topic.

There was also expansion of the data when integrating the quantitative and qualitative results. We noted the statistically significant increase in the clinical application subscore in the active learning format compared with the traditional format. Qualitative results suggest that a combination of increased internal motivation to learn about clinically relevant aspects of research, the requisite preparation for this format, and the increased interaction, albeit somewhat artificial in nature, of the active learning model may have improved the clinical application subscore for this format.

## Discussion

In this sequential, explanatory mixed-methods study comparing traditional and active learning journal club formats, we found that the active learning format was more effective in teaching clinical application concepts but found no differences across the 2 formats for the teaching of research methodology. When these findings were mixed with the qualitative results, we found that, similarly, few residents reported changes in behavior in reference to understanding of research methodology. However, perhaps, the more interactive nature of the active learning format when combined with elevated motivation to learn about clinically relevant topics led to significant changes in learning in the active learning format.

Few studies have compared different journal club formats in their effectiveness in teaching evidence-based practice (EBP). One study compared a traditional format with an evidence-based format and concluded that an EBP approach did not seem to improve emergency residents' critical appraisal skills.^12^ Another study compared an evidence-based journal club and traditional journal club and found that the traditional journal club was more effective in teaching the intense critique and analysis of an individual study, although overall critical appraisal skills did not differ across the 2 formats.^13^ Our findings may have differed from these because of different evaluation strategies. The first study gave both groups a factitious article to evaluate in an essay format before and after the 12-month study period and evaluated overall scores. The second study's outcomes were more similar to ours, with their “Study Analysis and Critique Section” grade analogous to our “Research Methodology” subscore and their “Study Conclusion” grade analogous to our “Clinical Application” subscore. They found that the mean “Study Conclusion” grade was higher in the EBP group but did not reach statistical significance (*p* = 0.07).

Our qualitative analysis revealed 4 primary themes of (1) the burden of preparation, (2) the importance of clinical relevance, (3) the predominance of passive listening in both formats, and (4) the value of faculty expertise in role-modeling evidence-based practice.

While residents acknowledged that advance preparation was important to arrive informed for the journal club, they found themselves limited by the competing clinical and professional demands of residency training. This finding echoes concerns about the feasibility of active learning, flipped classroom models in graduate medical education^14^ and highlights the need for curricula that effectively address the practical challenges inherent to graduate medical education.

Second, because clinical relevance acts as a strong internal motivator for engagement, journal club topics that are unrelated to residents' areas of interest may fail to inspire participation. In this study, residents expressed a consistent desire to have more input in selecting topics relevant to their future subspecialty. This aligns with previous research in which article selection was ranked as the largest contributing factor to journal club effectiveness.^15^ However, allowing residents to choose their own articles can result in the presentation of low-quality or inappropriate articles.^16^ In addition, given residents' diverse subspecialty interests, self-selection of topics may only boost motivation for a select few and is not believed to broadly increase attendance or engagement.^16^ Articles used in the journal club sessions were selected based on unique or relevant study designs and research methods felt by the group of experts to be of educational value to residents. If residents were permitted to select their own articles, that educational value may be lost. Therefore, while clinically relevant topics should be considered when designing journal club curricula, faculty input may be advisable to avoid the disadvantages mentioned. In addition, selected articles should either be foundational or published recently, such as in the past year, or considered highly impactful. Recent publications were preferred by residents in this study and previous studies^15^ and may compensate for a lack of topical relevance. Our results demonstrated a general lack of interest in research methodology among residents. Our quantitative analysis reveals that the difference in total scores observed in the active learning format was primarily driven by a greater understanding of clinical application rather than research methodology. Qualitative interviews suggest that this discrepancy stems from a lack of interest in research methodology, potentially attributable to low levels of intrinsic motivation.

Our third theme demonstrated that while the active learning format improved engagement, passive listening was nevertheless predominant and it tended to produce discussions that felt scripted and inorganic. This variability in the quality of discussion has been found by others who noted that poor group dynamics and noncontributing students could serve as a barrier for attempts to incorporate active learning pedagogies.^17^ These dynamics may then skew participation toward actively engaged students and can lead to unbalanced discussions. Similarly, the use of a discussion guide in the active learning format may have also served as a source of these inorganic discussions because it was possible that there was more of a perceived goal to address all the questions instead of encouraging an organic discussion around the article itself.

Faculty modeling should also be prioritized because residents found it highly valuable for role-modeling evidence-based practice. The shortage of trained and motivated faculty is a significant obstacle to integrating evidence-based practice into resident education.^18^ Therefore, it is crucial for future journal clubs to address this issue. Expanding on the role of faculty facilitators through online training or other methods of faculty development could be an effective solution.^18^ This training would enable facilitators to better fulfill key responsibilities, such as offering insightful commentary on study methodology, providing comprehensive summaries, and making connections to clinical practice. However, the feasibility of this strategy is reliant on the resources available to each residency program and institution.

Finally, SCLT may not have been the ideal theoretical framework. While SCLT is a valuable framework for teaching certain behaviors, particularly those that can be physically viewed and modeled, critical appraisal is a cognitive skill that requires comprehension and analysis of material. Instead, principles of andragogy seemed to predominate in our inductive thematic analysis, such as the importance of the need to know this material, as reflected in the theme on clinical relevance of articles.^19^ The principles of intrinsic motivation, readiness to learn, and “need-to-know” the material were likely crucial in the differences in methodology and clinical application subscores. Adult learners tend to have a problem-centered orientation, and so, having faculty experts model the application of information in a problem-centered approach may have been better aligned with adult learners' interests and goals.^19^ Others may similarly choose to frame the design of innovative journal club curricula with the 6 principles of andragogy in mind.

This study has several limitations that should be considered when interpreting the results. First, the additional 80 sister questions did not undergo their own pilot testing because of constraints in personnel and time. Therefore, while review by a senior educational analyst offered support for content validity in ensuring similarity of these questions with the original set, it is possible that there were minor differences that may have affected the results. Second, the sample size was small, given the single-center nature of this study, and varied between sessions, which may affect generalizability. However, we hoped that the qualitative data allowed for triangulation and transferability that would not be possible with either qualitative or quantitative data alone. The completion rates for pretests and posttests were notably lower in the later sessions, particularly for the traditional format, which could introduce bias in the results. Unfortunately, owing to the single-center nature of this study and the relatively small size of a neurology residency program, dividing the cohort into 2 groups for a randomized, controlled trial approach was not feasible. Future multicenter research studies may further validate these findings and explore alternative journal club formats. One potential model is the no-prep journal club, in which no preparation is necessary, and residents are asked to use their previous knowledge of study design to create their own study based on an initial prompt. Residents reported better comprehension of scientific literature after participating in a no-prep journal club and greater comfort with the analysis process.^20^ Because this format requires students to actively engage in study design, it may encourage greater engagement in discussions of research methodology.

This study also raises an important question about the role of journal clubs in residency education. Medical educators must remain open to the possibility that journal club may not be the most effective method of teaching evidence-based medicine to residents.^21^ In fact, there is evidence to suggest that evidence-based medicine (EBM) courses are most efficient for teaching EBM skills with or without an added journal club component.^22^ Based on the principles of andragogy, perhaps an educational experience grounded in clinical scenarios would increase motivation by emphasizing the immediate relevance of the learned material. Case-based learning, which involves presenting real patient scenarios as the context to explore relevant literature, may more effectively bridge the gap between research and clinical practice.^23^ Alternatively, future journal club curricula would likely benefit from introducing the curriculum with an overview of its purpose and its relevance in strengthening neurology residents' clinical foundation, regardless of subspecialty interest, thus aligning with principles of andragogy. Ultimately, there is likely no single correct strategy, and educators' decisions to explore alternative journal club formats, evidence-based medicine curricula or case-based learning to enhance critical appraisal skills will also depend on time, resources, and faculty available to facilitate the selected curricular approach.

This study underscores the need for effective educational strategies in teaching residents the skills of evidence-based practice. While the active learning journal club format showed promise in enhancing engagement and improving the skills of clinical application of research, challenges arose such as limited preparation time and the scripted nature of the discussions. Moving forward, exploring novel journal club formats such as the no-prep journal club could potentially mitigate these challenges, but other instructional strategies should be explored as well to improve learning of both research methodology and clinical application of research. Such efforts are critical in equipping residents with the essential skills to efficiently comprehend and effectively apply scientific research findings to clinical practice in an era of rapid neurology research advances.

## Glossary

EBM: evidence-based medicine
SCLT: social cognitive learning theory
